# OxLDL as a prognostic biomarker of plaque instability in patients qualified for carotid endarterectomy

**DOI:** 10.1111/jcmm.18459

**Published:** 2024-07-22

**Authors:** Agnieszka Woźniak, Joanna Satała, Paulina Gorzelak‐Pabiś, Agnieszka Pawlos, Marlena Broncel, Piotr Kaźmierski, Ewelina Woźniak

**Affiliations:** ^1^ Department of Internal Diseases and Clinical Pharmacology, Laboratory of Tissue Immunopharmacology Medical University of Lodz Lodz Poland; ^2^ Department of Vascular, General, and Oncologic Surgery Medical University of Lodz Lodz Poland

## Abstract

Atherosclerotic plaque instability increases the risk of stroke. As such, determining the nature of an instability atherosclerotic plaque may speed up qualification for carotid endarterectomy (CEA), thus reducing the risk of acute vascular events. The aim of the study was to determine the diagnostic value of oxidized LDL cholesterol (ox‐LDL), matrix metalloproteinase 9 (MMP‐9) and 8‐hydroxy‐2′‐deoxyguanosine (8‐OHdG) in serum as a prognostic markers of instability atherosclerotic plaques. Serum was collected from 67 patients who underwent CEA in accordance with the qualification criteria. The levels of ox‐LDL, MMP‐9 and 8‐OHdG were assessed by ELISA. The predictive value of the markers was determined based on an ROC curve, and the cut‐off points with the highest sensitivity and specificity were determined. Patients with unstable atherosclerotic plaque had significantly higher serum ox‐LDL, MMP‐9 and 8‐OHdG values. It was found that in patients before CEA, ox‐LDL >31.4 ng/mL was associated with an 82.5% probability of unstable atherosclerotic plaque, MMP‐9 >113.1 ng/mL with 78.6%, and 8‐OHdG >2.15 ng/mL with 64.7%. Multivariate regression analysis found ox‐LDL to be an independent factor associated with plaque instability. Patients with unstable plaques tend to have higher serum levels of ox‐LDL, MMP‐9 and 8‐OHdG compared to those with stable plaques. The optimal cut‐off point for ox‐LDL (AUC 0.86, *p* <0.0001) was 31.14 ng/mL, with 91.18% sensitivity and 78.79% specificity. The high sensitivity and specificity of ox‐LDL suggests that it can be used as an independent marker of plaque instability.

## 1 INTRODUCTION

The atherosclerotic plaque is stabilized by an extracellular matrix and dense fibrous cover. Together with the participation of macrophages and T lymphocytes, inflammatory processes in the plaque lead to the release of matrix metalloproteinases (MMPs) that digest collagen. This reduces the elasticity of the fibrous cover and thus the stability of the atherosclerotic plaque. An unstable plaque is more likely to demonstrate internal bleeding, which can lead to rupture. Rupture of atherosclerotic plaque is responsible for the overwhelming majority of acute events in plaque disruption or erosion with superimposed thrombosis.^1^ In addition, changes in the structure of blood vessels can alter the extracellular matrix.

Currently, a battery of imaging tests is needed to detect unstable lesions in atherosclerotic plaques, such as digital ultrasonographic diagnostic, computed tomography angiography or magnetic resonance angiography. Hence, there is a need to discover simpler methods that can be applied more widely for determining plaque instability.^2^

Accurate detection and determination of the type of atherosclerotic plaque may: first, speed up qualification for carotid endarterectomy (CEA) in asymptomatic patients with unstable atherosclerotic plaque; secondly, improve the prevention of acute cardiovascular events; thirdly, it may be helpful in preparing the patient for endarterectomy and avoiding its possible complications.

Recent research indicates that oxidized low‐density lipoproteins (ox‐LDL) play a key role in the formation of atherosclerotic lesions.^3^ Ox‐LDL indirectly affects the expression of MMPs and their activity within the atherosclerotic plaque.^4^ In addition, ox‐LDL can activate the monocyte chemotactic protein‐1, which causes endothelial damage and neovascularization.^5^

Among the 28 metalloproteinases studied to date, metalloproteinase 9 (MMP‐9) appears to be most frequently associated with cardiovascular pathology. An analysis of MMP‐9 gene polymorphisms found MMP‐9 to have potential clinical significance in the pathogenesis of atherosclerosis.^6^

One of the most studied biomarkers of oxidative DNA damage is 8‐hydroxy‐2′‐deoxyguanosine (8‐OHdG). It is an early biomarker of endothelial dysfunction, and high levels have been reported in atherosclerotic lesions of the aorta; in addition, the level of 8‐OHdG has been found to correlate with the number of vessels affected by atherosclerosis.^7^ Ox‐LDL reduces the level of enzymes involved in base excision repair,^8^ resulting in lowered removal of 8‐OH‐Gua adducts from DNA.^9^ Such events may hence contribute to the development of vascular diseases through the accumulation of DNA damage.^10^

The present study examines the relationship between serum levels of ox‐LDL, MMP‐9 and 8‐OHdG in patients with carotid stenosis before CEA and plaque morphology as determined by the surgeon during CEA.

While previous studies have examined the level of ox‐LDL and MMP‐9 in stable and unstable plaques, their cut‐off values for identifying unstable plaques have yet to be determined. Furthermore, the potential of ox‐LDL and 8‐OHdG to predict the stability of carotid atherosclerotic plaques also remains unstudied. Therefore, the aim of this paper is to evaluate the potential of ox‐LDL, MMP‐9 and 8‐OHdG as prognostic biomarkers of plaque instability before CEA.

## 2 MATERIALS AND METHODS

### 2.1 Patients and data collection

The study comprised 67 patients (35 male and 32 female) qualified consecutively for CEA according to 2017 European Society of Vascular Surgery (ESVS) guidelines. All patients showed symptomatic or asymptomatic unilateral carotid artery stenosis rate (50–90%) and were hospitalized at the Department of Vascular, General and Oncological Surgery, M. Copernicus Hospital, Lodz from July 2018 to October 2019.

The 2017 guidelines are identical to the current recommendations issued in 2023 by ESVS, both of which define significant carotid stenosis as ≥50% stenosis of the internal carotid artery. The severity of the stenosis is assessed using the North American Symptomatic Carotid Endarterectomy Trial (NASCET). Carotid stenosis is defined as *symptomatic* if associated with symptoms in the last 6 months, and *asymptomatic* if symptoms are absent or occurred greater than 6 months previously. CEA is recommended in asymptomatic patients with 60%–99% stenosis of the carotid artery who have abnormalities in imaging tests indicating a risk of stroke. CEA should be undertaken in symptomatic patients with 50%–99% stenosis.^11^

For inclusion in the study, the patients had to demonstrate more than 70% narrowing of the extracranial carotid artery due to atherosclerosis and be eligible for elective endarterectomy. However, the following exclusion criteria were applied: non‐vascular causes of ischemic stroke, previous haemorrhagic or lacunar stroke, brain diseases, treatment of atrial fibrillation, active inflammation or infection, autoimmune diseases, haematological and oncological diseases, severe renal or hepatic failure, myocardial infarction or surgical intervention within a year before inclusion, history of use of anti‐inflammatory or immunosuppressive drugs within 6 months before the examination, venous thrombosis, malnutrition, poisoning, or abuse of alcohol or other psychoactive substances. Any participants with the latter excluded from the study.

All CEA procedures were performed by the same vascular surgeon. During the Doppler ultrasound examination of the carotid arteries, the vascular surgeon paid particular attention to changes characterizing unstable atherosclerotic plaque, such as rupture or ulceration, the presence of internal bleeding or blood clots on its surface. Carotid stenoses were assessed according to the NASCET criteria.

Atherosclerotic plaques surgically removed from patients were examined macroscopically and microscopically at the Department of Vascular, General and Oncological Surgery, M. Copernicus Hospital in Lodz. The plaque classification was consistent with the guidelines of the American Heart Association (AHA) for advanced atherosclerotic lesions: type IV‐ atheroma with a confluent extracellular lipid core, type V‐ fibro‐atheroma, type VI‐ a complex plaque with some combination of disruptions of the lesion surface, hematoma or haemorrhage, and lumen thrombosis, type VII‐calcified plaque, type VIII‐fibrotic plaque without lipid core.

Whole blood was collected from the patients, and serum ox‐LDL, MMP‐9 and 8‐OHdG levels were determined. The blood was also tested for its full lipid profile (i.e. total cholesterol [TC], high‐density lipoprotein [HDL], non‐HDL, low‐density lipoprotein [LDL], triglycerides [TG], as well as lipoprotein (a), c‐reactive protein [CRP] and creatinine levels). Clinical data, including age, sex, history of ischemic strokes, diabetes, hypertension, obesity, smoking and presence of malignancies were also documented through a medical questionnaire/interview (Table 1).

**TABLE 1:** Clinical characteristics of the patients according to the presence of stable and unstable atherosclerotic plaque.

Parameter median/(IQR)	Total patients *n* = 67	Patients with stable atherosclerotic plaque *n* = 33	Patients with unstable atherosclerotic plaque *n* = 34	*p* value
Sex, *n* (%)				>0.05
Female	32 (46.8)	18 (54.5)	14 (41.2)	
Male	35 (52.2)	15 (45.5)	20 (58.8)	
Age (years)	71 (47; 91)	72 (47; 87)	68 (50; 91)	>0.05
Total cholesterol (mg/dL, normal range 130–200)	154 (95;276)	152 (95;243)	156 (103; 276)	>0.05
HDL‐C (mg/dL, normal range female >65, male >55)	45 (23;89)	48 (30;89)	43 (23; 70)	>0.05
Non‐HDL (mg/dL)	109 (52;236)	104 (52;184)	112 (54; 236)	>0.05
LDL‐C (mg/dL, normal range <55)	68 (15;188)	65 (15;144)	69 (28;188)	>0.05
TG (mg/dL, normal range 30–150)	137 (58;275)	128 (72; 275)	144 (58; 237)	>0.05
Lp(a) (nmol/L)	56 (0.5;305)	69 (3; 305)	46 (0.5; 202)	>0.05
hsCRP (mg/dL, normal range <5)	4 (0.3;46)	3 (0.3;9.7)	4 (0.4; 46)	>0.05
Creatinine (mg/L, normal range female <0.9, male <1.2)	0.9 (0.6;1.7)	0.9 (0.6; 1.3)	1 (0.6; 1.7)	>0.05
Pre‐existing medical conditions, *n* (%)				
Stroke or ischemic stroke	43 (73.1)	23 (69.7)	26 (76.5)	>0.05
Diabetes mellitus	15 (22.4)	7 (21.2)	8 (23.5)	>0.05
Arterial hypertension	52 (77.6)	28 (84.8)	24 (70.6)	>0.05
Obesity	5 (7.5)	1 (3.0)	4 (11.8)	>0.05
Smoking	29 (43.3)	14 (42.4)	15 (44.1)	>0.05
Cancer	3 (4.5)	2 (6.0)	1 (2.9)	>0.05

Abbreviations: HDL‐C, high‐density lipoprotein cholesterol; hsCRP, High sensitivity CRP; IQR, interquartile range, LDL‐C; low‐density lipoprotein cholesterol; TG, triglycerides.

The investigation was approved by the Bioethics Committee of the Medical University of Lodz number RNN/757/13/KB. Informed written consent was obtained from all participants. The study was completed in accordance with the ethical guidelines of the Declaration of Helsinki.

### 2.2 Study design

Twenty‐four hours before surgery, peripheral blood samples were collected in tubes containing EDTA (ethylenediaminetetraacetic acid). After centrifugation, the serum was frozen and stored at −80°C. Lipid profile (total cholesterol, HDL, non‐HDL, LDL‐C, TG) and creatinine level were determined by colorimetric assay. Immunoturbidimetric assay was applied to evaluate the concentration of both Lp (a) and CRP.

The levels of oxidized LDL cholesterol (ox‐LDL), matrix metalloproteinase 9 (MMP‐9) and 8‐hydroxy‐2′‐deoxyguanosine (8‐OHdG) in the serum were assessed by enzyme‐linked immunosorbent assay (ELISA) using an ELISA ST‐360 microplate reader (450 nm). ELISA was performed using kits according to the manufacturer's protocols: ox‐LDL (Immunodiagnostik AG, Germany), MMP‐9 (Cloud‐Clone Corp., USA) and 8‐OHdG (Immunodiagnostik AG, Germany). The detection range of MMP‐9 was 0.156–10 ng/mL. The range of ox‐LDL was 41–2261 ng/mL and the range for 8‐OHdG was 0.125–10 ng/mL.

### 2.3 Statistical analyses

The relationships between pairs of groups were tested with the Mann–Whitney test (non‐normal distribution). Multivariable logistic regression was also generated, using plaque instability as an outcome and the selected variables, viz. ox‐LDL, MMP‐9 and 8‐OHdG and comorbidities, as predictors The predictive power was evaluated by receiver operating characteristics (ROC) and area under the curve (AUC). The optimal cut‐off point in the ROC analysis was chosen with the ‘CutoFinder’ (http://molpath.charite.de/cut‐off) online tool using ‘Manhattan distance’ (Institut für Pathologie, Charité‐Universitätsmedizin Berlin, Berlin, Germany). Statistical analyses were performed with the GraphPad Prism 9.0 (GraphPad Software, San Diego, California, United States). All tests were considered significant at a *p*‐value below 0.05.

## 3 RESULTS

### 3.1 Baseline characteristics of patients

Among the 67 enrolled patients, 35 (52.2%) were men, and 32 (46.8%) were women. The median age was 71 years (IQR, 46–91 years). Stable atherosclerotic plaque was diagnosed in 33 (49.3%) patients: 15 (45.5%) men and 18 (54.5%) women. Unstable atherosclerosis plaque was diagnosed in 34 (50.7%) patients: 20 (58.8%) men and 14 (41.2%) women. No sex dominance was observed between groups (*p* > 0.05).

Data regarding the lipid profile (total cholesterol, HDL, non‐HDL, LDL‐C, TG), lipoprotein (a), hsCRP and creatinine are given in Table 1. No statistically significant differences in lipid profile were found between patients with stable and unstable atherosclerotic plaques. The risk factors for formation of atherosclerotic plaques observed in the patients are also presented in Table 1.

### 3.2 Among patients qualified for CEA, serum levels of ox‐LDL, MMP‐9 and 8‐OHdG are significantly higher in those with unstable atherosclerotic plaque compared to those without

**FIGURE 1:**
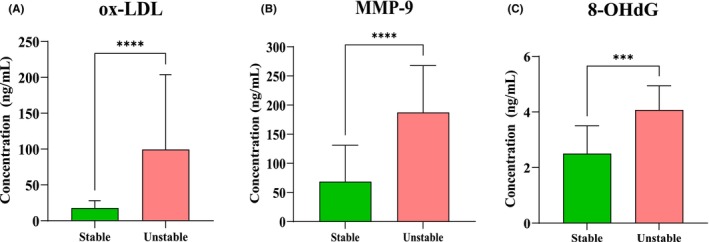
Ox‐LDL (A), MMP‐9 (B) and 8‐OHdG (C) serum levels in patients qualified for CEA with stable and unstable atherosclerotic plaque (for ox‐LDL and MMP‐9 *p* < 0.0001; 8‐OHdG *p* < 0.001).

Patients with unstable atherosclerotic plaques demonstrated higher ox‐LDL serum levels (88.01 [41–185] ng/mL) than those with stable atherosclerotic plaque (17.73 [5–28]ng/mL; *p* < 0.0001) (Figure 1A).

Patients with unstable atherosclerotic plaques had higher MMP‐9 serum levels (187.3 [126–268] ng/mL) than those with stable atherosclerotic plaque (68.74 [45–131]ng/mL; *p* < 0.0001) (Figure 1B).

Patients with unstable atherosclerotic plaques had higher 8‐OHdG serum levels (4.1^2^, ^3^, ^4^, ^5^ ng/mL) than those with stable atherosclerotic plaque (2.5 [0.8–4] ng/mL; *p* < 0.001) (Figure 1C).

### 3.3 Serum levels of ox‐LDL, MMP‐9 and 8‐OHdG are higher in patients with unstable atherosclerotic plaque compared to those without, regardless of stroke occurrence

Both stroke and non‐stroke patients with unstable atherosclerotic plaques had higher serum ox‐LDL levels (for stroke patients: 94.51 [39–172]ng/mL; non‐stroke patients: 101.8 [51–1426]ng/mL; *p* < 0.0001) compared to either stroke or non‐stroke patients with stable plaque (for stroke patients: 16.16 [5–25]ng/mL; non‐stroke patients: 21.67 [8–28]ng/mL; *p* < 0.001), Table 2.

**TABLE 2:** Serum levels of ox‐LDL, MMP‐9 and 8‐OHdG in stroke/non‐stroke patients with stable and unstable atherosclerotic plaque qualified to CEA.

Parameter	Ox‐LDL	MMP‐9	8‐OHdG
Stroke patients with stable atherosclerotic plaque (*n* = 23)	16.16 (5; 25)	68.74 (43; 87)	2.53 (0.8; 3.6)
Stroke patients with unstable atherosclerotic plaque (*n* = 26)	94.51 (39; 172)	175.2 (133; 261)	3.9 (2.3; 5)
*p* value	**<0.0001**	**<0.0001**	**<0.01**
Non‐stroke patients with stable atherosclerotic plaque (*n* = 8)	21.67 (8; 28)	65.94 (42; 168)	2.21 (1; 3.5)
Non‐stroke patients with unstable atherosclerotic plaque (*n* = 8)	101.8 (52; 1426)	217.9 (123; 304)	4.2 (1.9;4.6)
*p* value	**<0.001**	**<0.05**	**<0.05**

Bold values denote statistical significance at the p < 0.05 level.

Both stroke and non‐stroke patients with unstable plaques had higher serum MMP‐9 levels (for stroke patients: 175.2 [133–261]ng/mL; non‐stroke patients: 217.9 [123–304]ng/mL; *p* < 0.0001) than those without stable atherosclerotic plaque (for stroke patients: 68.74 [43–87]ng/mL; non‐stroke patients: 65.94 [42–168]ng/mL; *p* < 0.001), Table 2.

Stroke and non‐stroke patients with unstable plaques had higher serum 8‐OHdG levels (for stroke patients: 3.9 [2.3–5]ng/mL; non‐stroke patients: 4.2 [1.9–4.6]ng/mL; *p* < 0.01) compared to those with stable plaque (for stroke patients: 2.53 [0.8–3.6]ng/mL; non‐stroke patients: 2.21 [1–3.5]ng/mL; *p* < 0.05), Table 2.

### 3.4 Serum levels of ox‐LDL, MMP‐9 and 8‐OHdG are higher in patients with unstable atherosclerotic plaques compared to those without, regardless of serum Lp(a) levels

Regardless of Lp(a) level, patients qualified for CEA with unstable atherosclerotic plaques demonstrated higher serum ox‐LDL levels (both patients with Lp(a) ≥125 nmol/L (59 [34; 83]ng/mL) and Lp(a) <125 nmol/L) (109.2 [77; 250]ng/mL); (*p* < 0.05) than patients with stable atherosclerotic plaque (in patients with Lp(a) ≥125 nmol/L: 20.88 [2; 29]ng/mL; in patients with Lp(a) <125 nmol/L: 18.52 [7; 31]ng/mL; *p* < 0.0001, Table 3).

**TABLE 3:** Ox‐LDL, MMP‐9 and 8‐OHdG concentrations in the serum of patients, taking into account the level of Lp(a) in the serum of patients qualified for CEA.

Parameter	Ox‐LDL	MMP‐9	8‐OHdG
Lp(a) ≥125 nmol/L; patients with stable atherosclerotic plaque (*n* = 7)	20.88 (2; 29)	72.26 (43; 138)	2.72 (2.4; 3.6)
Lp(a) ≥125 nmol/L; patients with unstable atherosclerotic plaque (*n* = 3)	59 (34; 83)	122.9 (122; 150)	4.17 (3.7; 4.3)
*p* value	**<0.05**	>0.05	**<0.05**
Lp(a) <125 nmol/L; patients with stable atherosclerotic plaque (*n* = 22)	18.52 (7; 31)	67.87 (44; 110)	2.36 (0.9; 3.4)
Lp(a) <125 nmol/L; patients with unstable atherosclerotic plaque (*n* = 27)	109.2 (77; 250)	198.2 (134; 271)	3.78 (2.2; 4.9)
*p* value	**<0.0001**	**<0.0001**	**<0.01**

Bold values denote statistical significance at the p < 0.05 level.

Patients with unstable atherosclerotic plaques exhibited higher serum levels of MMP‐9 (both patients with Lp(a) ≥125 nmol/L (122.9 [122; 150]ng/mL) and Lp(a) <125 nmol/L (198.2 [134; 271]ng/mL)) than those with stable atherosclerotic plaque: in patients with Lp(a) ≥125 nmol/L: 72.26 [43; 138] ng/ml; in patients with Lp(a) <125 nmol/L: 67.87 [44; 110]ng/mL; (*p* < 0.0001, Table 3).

Patients with unstable atherosclerotic plaques demonstrated higher serum levels of 8‐OHdG that is, both patients with Lp(a) ≥125 nmol/L (4.17 [3.7; 4.3]ng/mL) and Lp(a) <125 nmol/L (3.78 [2.2; 4.9]ng/mL); (*p* < 0.05), compared to those with stable atherosclerotic plaque: in patients with Lp(a) ≥125 nmol/L: 2.72 [2.4; 3.6]ng/mL; in patients with Lp(a) <125 nmol/L: 2.36 [0.9; 3.4]ng/mL; (*p* < 0.01, Table 3).

### 3.5 ROC curve analyses

**FIGURE 2:**
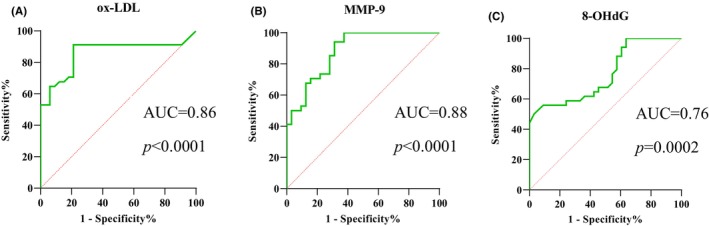
Receiver operating characteristic (ROC) curves showing ox‐LDL (A) MMP‐9 (B) and 8‐OHdG (C) serum levels in patients.

An ROC curve was plotted to determine the potential of ox‐LDL, MMP‐9 and 8‐OHdG serum levels to predict the instability of atherosclerotic plaque. Figure 2A shows the ROC curve for ox‐LDL (AUC = 0.86, *p* < 0.0001); the optimal cut‐off point for this parameter was 31.14 ng/mL, with a sensitivity of 91.18% and specificity of 78.79%. Figure 2B shows the ROC curve for MMP‐9 (AUC = 0.88, *p* < 0.0001); the optimal cut‐off point for this parameter was 113.1 ng/mL, with a sensitivity of 85.29% and specificity of 71.88%. Finally, Figure 2C shows the ROC curve for 8‐OHdG (AUC = 0.76, *p* = 0.0002); the optimal cut‐off point was 2.15 ng/mL, with a sensitivity of 82.35% and specificity of 42.42%.

### 3.6 Logistic regression analysis indicates that ox‐LDL is an independent predictor of plaque instability

**FIGURE 3:**
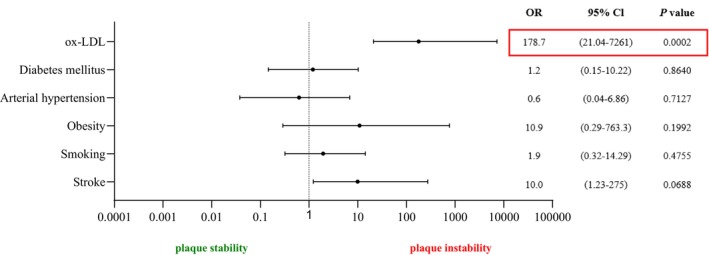
Logistic regression analysis regarding potential risk factors for unstable atherosclerotic plaque in patients qualified for CEA.

A logistic regression model (Figure 3) was generated to evaluate potential risk factors for unstable atherosclerotic plaque, that is, stroke, diabetes mellitus, arterial hypertension, obesity, smoking and ox‐LDL as predictor. Following this, the marker with the highest sensitivity and specificity, ox‐LDL, was subjected to logistic regression analysis. The logistic regression model indicated that ox‐LDL is an independent factor and is not influenced by other occurring factors.

### 3.7 Logistic regression analysis indicates that the combination of ox‐LDL and MMP‐9 provides a stronger model of atherosclerotic plaque instability

**FIGURE 4:**
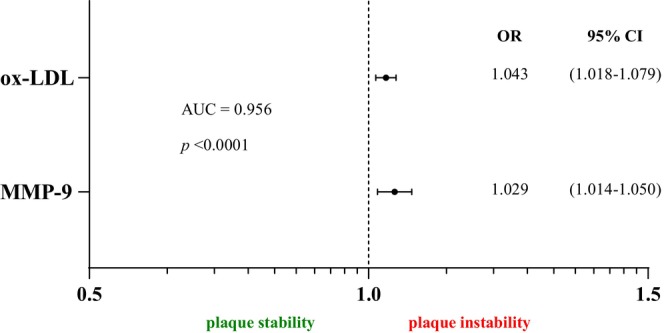
Multivariate regression model for predicting carotid plaque instability.

A logistic regression model (Figure 4) was generated to assess the potential risk of unstable atherosclerotic plaque for the combination of the two markers with the highest sensitivity and specificity. These markers were then subjected to logistic regression analysis. The logistic regression model indicated that ox‐LDL combined with MMP‐9 had greater predictive power (AUC = 0.956, *p* < 0.0001).

Moreover, Spearman's correlation analysis was performed for all three markers with lipid parameters, CRP, creatinine and age, which indicates that the only correlation that was obtained was the positive correlation between ox‐LDL and MMP9 (*p* = 0.57). There is no correlation between markers and other factors. There was no correlation between the three markers and lipid profile or age (Supplement 1).

## 4 DISCUSSION

Patients qualified for CEA with unstable atherosclerotic plaque were found to exhibit significantly higher oxidized LDL (ox‐LDL) levels than those with stable plaques, suggesting that plaque ox‐LDL in serum is associated with plaque instability. Importantly, no significant differences in LDL level were noted between these two groups, indicating that ox‐LDL has clinical potential as a marker in these patients. Moreover, a strong correlation was noted between ox‐LDL level and the likelihood of unstable atherosclerotic plaque in patients qualified for CEA. The plotted ROC curve indicates that a patient with an ox‐LDL serum level >31.14 ng/mL has an 82.5% probability of unstable plaque. A patient below this value has an 89.6% probability of having a stable plaque.

In addition, elevated levels of ox‐LDL (as well as MMP‐9 and 8‐OHdG) were noted in the serum of patients qualified for CEA, regardless of lipoprotein (a) level, an independent factor and predictor of stroke (>125 nmol/L).^12^ This further confirms the clinical value of ox‐LDL determination.

In addition, all three markers were elevated in patients with unstable atherosclerotic plaques, regardless of whether they had previously suffered a stroke. Suzue et al. report an increase in both ox‐LDL and MMP‐9 in patients with unstable plaques after stroke.^13^

Ox‐LDL stimulates macrophages; these act as sources of MMPs, which are responsible for the degradation of connective tissue elements.^4^ MMPs also play a significant role in the degradation of elastin fibres. These, in turn, form connections with receptors on the surface of vascular smooth muscle cells, contributing to their differentiation. Subsequently, smooth muscle cells and macrophages demonstrate increased MMP expression, which can destabilize the atherosclerotic plaque in the vascular wall. Particularly high MMP levels are observed in the area of foam cell accumulation.^14^

One of the most widely‐documented metalloproteinases in atherosclerotic plaques is MMP‐9, which plays a key role in the stability of the plaque.^15^ MMP‐9 has also been found to be distributed in the medullary and fibrous cap regions of the plaque, with significantly higher levels noted in unstable plaques.^16^

Interestingly, Olson et al. report no relationship between plasma MMP‐9 concentration and the stability of the atherosclerotic plaque in the carotid artery; however, a significant relationship was noted in plaques obtained from the femoral arteries.^17^

Our present findings indicate significantly higher serum concentrations of MMP‐9 in patients with unstable atherosclerotic plaques than in those with stable atherosclerotic plaques. The plotted ROC curve, and hence the predictive value of the index, indicated a 78.6% probability that a patient with an MMP‐9 serum level >113.1 ng/mL on admission has an unstable plaque. A patient below this cut‐off value has an 82.7% probability of having a stable plaque.

This is the first finding to suggest that 8‐OHdG may be a marker of atherosclerotic plaque stability. However, previous studies confirm high levels of 8‐OHdG in patients with cardiovascular diseases. A prospective study by Brea et al. showed that patients who experienced recurrent stroke or cardiac death had higher levels of 8‐OHdG.^18^

An initial meta‐analysis found 8‐OHdG levels to be higher in patients with atherosclerotic cardiovascular disease (ASCVD), coronary artery disease (CAD) and other types of atherosclerotic processes, such as peripheral arterial or carotid atherosclerosis than controls.^7^ Higher serum 8‐OHdG levels have also been noted in asymptomatic patients with carotid plaque than in control patients without cardiovascular disease.^19^

Our present findings indicate significantly higher serum concentrations of 8‐OHdG in patients with unstable atherosclerotic plaques than in patients with stable atherosclerotic plaques. Based on the plotted ROC curve, and hence the predictive value of the index, it can be seen that a patient qualified for CEA, with serum 8‐OHdG >2.15 ng/mL has a 64.7% probability of an unstable plaque, while a patient below this value (i.e. 8‐OHdG <2.15 ng/mL) has an 82.3% probability of having a stable plaque. 8‐OHdG is not an independent factor.

The ROC curve analysis played a key role in evaluating the potential of ox‐LDL, MMP‐9 and 8‐OHdG to predict vulnerable carotid plaque in patients. However, the AUC was above 0.75 in each index, which may indicate that the use of these three indexes will be of value in diagnosing atherosclerotic unstable plaques.

While all selected biomarkers demonstrated high AUC values, the best biomarker of atherosclerotic plaque instability in our opinion, due to the highest sensitivity and specificity, is ox‐LDL; in addition, ox‐LDL appears to be an independent factor, based on the logistic regression model. Ox‐LDL used as a marker also has several other advantages, most importantly, it is a universal indicator that does not depend on comorbidities and other tested parameters, and laboratory testing is less expensive—compared to MMP‐9 or the much more expensive 8‐OHdG. Increased levels of ox‐LDL activate intracellular signalling pathways that lead to an increase in the level of adhesion molecules, the release of pro‐inflammatory cytokines and metalloproteinases, consequently stimulating angiogenesis; as such, it may be suitable as an early detection marker.

The AUC analysis indicates that ox‐LDL and MMP‐9, have greater power when tested in combination. However, such testing would also generates additional costs, and ox‐LDL determination alone appears sufficient to predict unstable atherosclerotic plaque.

In contrast, while 8‐OHdG demonstrates high sensitivity, it is also characterized by relatively low specificity; as such, it should not be used as a sole biomarker of plaque instability.

Such testing may play an important role in preventing, cardiovascular events in patients with unstable atherosclerotic plaque. Accelerating qualification for endarterectomy, especially in asymptomatic patients and avoiding possible post‐procedure complications is of considerable value to both clinician and patient.

## 5 CONCLUSION

Patients with unstable carotid plaques have higher levels of ox‐LDL, MMP‐9 and 8‐OHdG than patients with stable plaques. Of these, the best prognostic independent marker for plaque instability in patients qualified for CEA appears to be ox‐LDL. Elevated levels of ox‐LDL, as well as MMP‐9 and 8‐OHdG, were noted in the serum of patients qualified for CEA. However, of these, ox‐LDL is the most desirable marker of plaque instability for use in everyday clinical practice due to its high sensitivity and specificity, and could be very helpful in identifying patients with unstable carotid atherosclerotic plaques and qualifying them for endarterectomy.

## 6 LIMITATIONS

There are several limitations to this study. Firstly, the stability of the isolated atherosclerotic plaque was not confirmed histopathologically. Secondly, the study does not include any data from ultrasound examinations of the carotid arteries; this however, may be a useful goal of future studies aimed at checking the correlation of the examined biomarkers with ultrasound parameters. Finally, it does not include any information on postoperative complications in these patients (such as stroke) that could be associated with unstable atherosclerotic plaque.

## AUTHOR CONTRIBUTIONS

**Agnieszka Woźniak:** Data curation (equal); formal analysis (equal); project administration (equal); resources (equal); software (equal); supervision (equal); writing – original draft (equal); writing – review and editing (equal). **Joanna Satała:** Formal analysis (equal); software (equal). **Paulina Gorzelak‐Pabiś:** Conceptualization (equal); data curation (equal); formal analysis (equal); investigation (equal); project administration (equal); resources (equal); software (equal); visualization (equal); writing – review and editing (equal). **Agnieszka Pawlos:** Visualization (equal); writing – review and editing (equal). **Marlena Broncel:** Conceptualization (equal); data curation (equal); investigation (equal); project administration (equal); software (equal); visualization (equal). **Piotr Kaźmierski:** Investigation (equal); software (equal). **Ewelina Woźniak:** Conceptualization (equal); formal analysis (equal); project administration (equal); resources (equal); software (equal); visualization (equal); writing – original draft (equal); writing – review and editing (equal).

## CONFLICT OF INTEREST STATEMENT

The authors declare that they have no known competing financial interests or personal relationships that could have appeared to influence the work reported in this paper.

## Supporting information


**Supplement 1**.

## Data Availability

The data that support the findings of this study are available from the corresponding author upon reasonable request.
