# Supplementary material for: OxLDL as a prognostic biomarker of plaque instability in patients qualified for carotid endarterectomy
Source: J Cell Mol Med. 2024 Jul 22;28(14):e18459. doi: 10.1111/jcmm.18459 (PMC11263466; doi:10.1111/jcmm.18459)
Supplement: Supplementary file 1 — Supplement 1. [file JCMM-28-e18459-s001.docx]

**Supplement 1.**

Spearman's correlation analysis was performed for all three markers with lipid parameters, CRP, creatinine and age, which indicates that the only correlation that was obtained was the positive correlation between ox-LDL and MMP9 (*p* = 0.57). There is no correlation between markers and other factors. There was no correlation between the three markers and lipid profile or age.
